# Protecting the Feet

**Published:** 1878-02

**Authors:** 


PROTECTING the FEET

from the dampness of the decks is an indispensable item of health and comfort on ship-board as the boards are seldom dry for two hours at a time in any voyage. Thick soled shoes and woolen hose should be worn at all times while at sea. Much has been said and written in praise of the

